# Post-exposure prophylaxis for SIV revisited: Animal model for HIV prevention

**DOI:** 10.1186/1742-6405-3-29

**Published:** 2006-11-28

**Authors:** Peter Emau, Yonghou Jiang, Michael B Agy, Baoping Tian, Girma Bekele, Che-Chung Tsai

**Affiliations:** 1 Washington National Primate Research Center, University of Washington, Box 357330 Health Sciences Building, Seattle, Washington 98195, USA

## Abstract

**Background:**

A 4-week, uninterrupted treatment with 9-(2-phosphonyl-methoxypropyly)adenine (PMPA, commonly called tenofovir) completely prevents simian immunodeficiency virus (SIV_mne_) infection in cynomolgus macaques if treatment begins within 24 hours after SIV_mne _inoculation, but is less effective if treatment is delayed or duration of treatment is shortened. Critical factors for efficacy include timing and duration of treatment, potency of antiretroviral drug and a contribution from antiviral immune responses. Therefore, we evaluated the impact of one or more treatment interruptions plus SIV_mne _re-exposures on efficacy of PMPA treatment to prevent SIV_mne _infection in cynomolgus macaques. We also evaluated whether macaques with pre-existing SIV immune responses show increased efficacy of treatment. Eight PMPA-treated, virus-negative and seronegative macaques, and five PMPA-treated, virus-negative but weakly or strongly seropositive macaques were re-inoculated with SIV_mne _and treated with PMPA starting 24 hr post inoculation. Thereafter, they received either a 5-week treatment involving one interruption plus one SIV_mne _challenge or a 10-week treatment involving six interruptions plus six SIV_mne _challenges early during treatment. Parameters measured were plasma SIV RNA, SIV-antibody response, CD4+ T lymphocyte subsets and *in vivo *CD8+ cell-suppression of virus infection.

**Results:**

All seronegative macaques developed persistent antibody response beginning 4 to 8 weeks after stopping PMPA-treatment in absence of viremia in a majority of macaques and coinciding with onset of intermittent viremia in other macaques. In contrast, all weakly or strongly seropositive macaques showed immediate increase in titers (> 1600) of SIV antibodies, even before the end of PMPA-treatment, and in absence of detectable viremia. However, in vivo CD8+ -cell depletion revealed CD8 cell-suppression of viremia and persistence of virus in the macaques as long as 2 years after PMPA-treatment, even in aviremic macaques. Unlike untreated macaques, a treated macaque controlled viral replication and blocked CD4+ T cell depletion when challenged with a heterologus chimeric SIV/HIV-1 virus called SHIV_89.6P._

**Conclusion:**

A single interruption plus one SIV_mne _challenge was as sufficient as six interruptions plus six SIV_mne _challenges in reducing efficacy of PMPA, but results in long-term persistence of virus infection suppressed by CD8+ cells. Efficacy of PMPA treatment was highest in macaques with pre-existing SIV immune responses.

## Background

Despite expanding use of antiretroviral therapy (HAART) [1], which has clearly extended lives of persons infected with human immunodeficiency virus (HIV) [2,3], the virus continued to spread worldwide at nearly 5 million new infections in 2005 [4]. Therefore, there is a need to revisit proven strategies of HIV prevention with a goal to understand their limitations and maximize their effectiveness. A strategy of post exposure prophylaxis (PEP) using highly potent antiretroviral drugs is effective in preventing human immunodeficiency virus (HIV) transmission in clinical situations where treatment can be started immediately after virus exposure. For example, in preventing vertical transmission of HIV from HIV-infected mothers to their infants [5,6], following occupational exposure to HIV in blood and body fluids from HIV-infected persons [7,8] or following sexual assault or intravenous drug use [9,10]. Nevertheless, major barriers to the success of the program are uncertainty as to the time of virus exposure and poor compliance in completing treatment regimen, partly due to drug toxicity [9-11]. Therefore, a regimen of pre-exposure prophylaxis is being evaluated for preventing HIV infection in high-risk, HIV-negative persons, such as sex workers whereby highly potent antiviral drugs are taken before high-risk behavior [9,12]. The rationale for pre or post exposure prophylaxis is that after HIV exposure there is a brief window of time, before the virus spreads systemically throughout the lymphoid organs, when initiating potent antiretroviral therapy might prevent or modify viral replication. In clinical settings in which compliance to treatment is poor and a potential exist for re-exposures to virus, PEP should at least reduce virus to a level sufficient to stimulate protective immune response such as antiviral CD8+ cells and thus reduce the probability of establishing persistent, productive infection. The efficacy of such regimen depends on the timing and duration of treatment, use of highly potent antiretroviral drugs and by immune responsiveness of the host [13,14].

We showed previously that early treatment with [(R)-9-(2-phosphonylmethoxypropyl)adenine] (PMPA) can completely prevent SIV_mne _infection in cynomolgus macaques if treatment begins within 24 hours post-inoculation (p.i.) and is continued uninterrupted for 4 weeks, but is less effective if the initiation of treatment is delayed or if the duration of treatment was shortened [15,16]. The highest efficacy achieved required an effective regimen (i.e. 24-hour p.i., 28-day treatment) that maintained therapeutic levels of PMPA to block the spread of virus, perhaps with a contribution from antiviral immune response. The less effective regimens such as delayed initiation of PMPA treatment or shortened duration of PMPA-treatment revealed the contribution of immune response to efficacy. These regimens resulted in either delayed establishment of virus infection or induced viral control by macaques leading to transient infections [15]. Additionally, although the PMPA-protected macaques remain free of detectable virus or SIV antibody response, they show partial resistance to challenges with homologous or heterologous SIV [17,18]. Even after the onset of SIV infection in macaques, the initiation of PMPA treatment during primary infection can induce immune suppression of SIV infection [19-23] mediated by CD8+ lymphocytes [18,24]. These studies indicated that regimens of early PEP with PMPA induce antiviral immune responses in macaques to control subsequent virus infection. The CD8+ lymphocyte-mediated control of virus replication is also a major mechanism by which early antiretroviral treatment of acute HIV-1 infection induces immune control of viral replication in HIV-1 infected persons [25]. The high potency of early PMPA treatment may be due to the rapid intracellular phosphorylation of PMPA into its active metabolites and the long half-life of these active metabolites [26], which disrupts the replication cycle of virus.

In the present study, we revisited our post exposure chemoprophylaxis against acute SIV_mne _infection in cynomolgus macaques [15-17] as an animal model for studying factors involved in efficacy of early antiretroviral intervention in HIV infection. We evaluated the impact of one or more interruptions of PMPA-treatment plus re-exposures to virus on the prevention of SIV_mne _infection and induction of CD8+ lymphocyte-mediated suppression of viremia in cynomolgus macaques. We also evaluated whether the efficacy of early PMPA treatment is greatest in macaques with pre-existing immune response to SIV.

## Results

### Efficacy of PMPA-treatment

**Figure 1:**
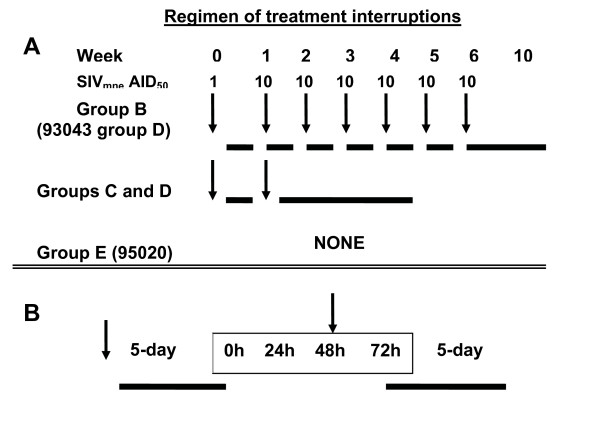
**A**. A schematic drawing SIV_mne _inoculations and treatment regimens of different groups. Arrows show schedule of SIV_mne _inoculation. The solid horizontal bar indicate schedule of daily PMPA treatment (30 mg/kg, subcutaneous). AID_50 _is 50% animal infectious dose of the SIV_mne _used in the studies. One AID_50 _of the SIVmne stock was approximately10 50% tissue culture infectious dose (TCID_50_) by intravenous inoculation, and 100 TCID_50 _by intrarectal inoculation. Macaques in groups B (plus macaque 93043 in group D) received a 10-week PMPA treatment beginning 24 hours after virus inoculation, including treatment interruption plus SIV_mne _re-inoculation with 10 AID_50 _SIV_mne _at weekly intervals during the first six weeks of treatment. Macaques in groups C and D received a 5-week PMPA treatment starting 24 hours after virus inoculation, including one treatment interruption plus SIV_mne _re-inoculation with 10 AID_50 _SIV_mne _at week 1 of treatment. Macaque 95020 (group E) was untreated. **B**. The exact timing of events during the 72 hour interruption, including timing of SIV_mne _re-inoculation during the treatment interruption. Treatment was initiated 24 hours after SIVmne inoculation, then continued for 5 days. Thereafter, treatment was withheld for 72 hours, during which macaques were re-inoculated with SIV_mne _at 48 h and re-started on treatment at 72 h. After the last inoculation, treatment was continued uninterrupted for 28 days. Note that the during the treatment interruption, the 48-hour interval between the end of treatment and SIV_mne _re-inoculation was approximately one half-life of PMPA active metabolites in resting lymphocytes or three times the half-life in activated lymphocytes [26].

The study subjects, called PEP-macaques, were fourteen cynomolgus macaques with a prior history of post exposure prophylaxis (PEP) of SIV_mne _infection using tenofovir (PMPA) or adefovir (PMEA) [15-17]. When grouped by serologic outcome at the start of the present study (after 4–5 years of follow-up study); the study subjects consisted of eight virus-negative and SIV antibody-negative (V^-^Ab^-^) PEP-macaques, four virus-negative but weak SIV-antibody positive (V^-^Ab^±^) PEP-macaques, and two virus-negative and strongly SIV antibody positive (V^-^Ab^+^) PEP-macaques. To facilitate comparison of data for individual macaques the history of individual macaques and their study groups are shown in Table 1. The schedule and regimens of treatment interruptions and SIV_mne _inoculations are shown schematically in FIG 1.

**Table 1:** Macaque history and regimen of post exposure prophylaxis.

**Group**	**Macaque**	**Treatment regimens**		
		Uninterrupted PEP^1^	Outcome after 4–5 years.^2^	Second PEP regimen^3^

A	98021	Naïve	V^-^Ab^-^	Untreated
	98034	Naïve	V^-^Ab^-^	Untreated
	98035	Naïve	V^-^Ab^-^	Untreated

B	93192	24 h pi 28 d	V^-^Ab^- ^(5)	10 wk, 6 ti
	93208	24 h pi 28 d	V^-^Ab^- ^(5)	10 wk, 6 ti
	95025	24 h pi 28 d	V^-^Ab^- ^(4)	10 wk, 6 ti
	95044	24 h pi 28 d	V^-^Ab^- ^(4)	10 wk, 6 ti

C	93194	-48 h pi 28 d	V^-^Ab-(5)	5 wk, 1 ti
	95054	24 h pi 28 d	V^-^Ab^- ^(4)	5 wk, 1 ti
	93217	24 h pi 28 d	V^-^Ab^- ^(5)	5 wk, 1 ti
	93193	4 h pi 28 d	V^-^Ab^- ^(5)	5 wk, 1 ti

D	M94312	24 h pi 28 d	V^-^Ab^± ^(4)	5 wk, 1 ti
	M95033	72 h pi 28 d	V^-^Ab^± ^(4)	5 wk, 1 ti
	95053	24 h pi 10 d	V^-^Ab^± ^(4)	5 wk, 1 ti
	93040	-48 h pi, 28 d +SIV challenge	V^-^Ab^+ ^(5)	5 wk, 1 ti
	93043	-48 h pi, 28 d +SIV challenge	V^-^Ab^+ ^(5)	10 wk, 6 ti

E	95020	24 h pi 10 d	V^-^Ab^± ^(4)	NONE

All macaques in this study were cynomolgus macaques (*Macaca fasicularis*).

^1^PEP, post-exposure chemoprophylaxis; uninterrupted PEP (1^st ^PEP) was performed using SIV_mne _infection and PMPA or PMEA as described in studies already published [15-17] 4–5 years prior to the present study (2^nd ^PEP). The uninterrupted PEP regimen such as 24 h pi 28 d indicates 24 hours post inoculation and 28 days of treatment.

^2^V^-^Ab^- ^indicates virus-negative and antibody-negative; V^-^Ab^± ^indicates virus-negative and weakly seropositive, V^-^Ab^+ ^indicates virus-negative and strongly seropositive. The numbers in brackets refers to years after uninterrupted PEP for follow-up of each macaque. Macaques 93040 and 93043 had been challenged with SIV_smmpBj14 _at 12 months after uninterrupted PEP as described previously [17] prior to being used in the present study.

^3^For second PEP regimen, all previously SIV-exposed and PMPA-treated macaques were divided into groups (B – E) based in the pre-existing SIV-antibody response. Group A were three naïve macaques that served as infection controls for SIV_mne _virus stock. Macaques in group B and one macaque (93043) in group D were given a 10-week (10 wk) PMPA treatment that included weekly treatment interruption(ti) plus SIV_mne _challenge for the first six weeks of treatment (i.e. 10 wk, 6 ti). Macaques in groups C and D were given a 5-week (5 wk) PMPA treatment that included a single treatment interruption (ti) plus SIV_mne _challenge during the first week of treatment (i.e. 5 wk, 1 ti). Macaque 95020 in group E received uninterrupted PMPA PEP but not the second PMPA PEP and remained virus-negative and weakly seropositive throughout the follow-up studies.

Briefly, group A were three naïve, untreated cynomolgus macaques were infection controls for SIV_mne _inoculum. Group B were four V^-^Ab^-^PEP-macaques that were given a 10-week PMPA-treatment beginning 24 hours after intravenous SIV_mne _inoculation, including treatment interruption and SIV_mne _challenge at weekly intervals during the first six weeks of treatment. Group C were four V^-^Ab^- ^PEP macaques that were given a 5-week PMPA-treatment beginning 24 hours after intravenous SIV_mne _inoculation, including one treatment interruption plus SIV_mne _challenge at week 1 of treatment. Group D was one V^-^Ab^+ ^-PEP macaque that received a treatment regimen similar to group B and three V^-^Ab^± ^and one V^-^Ab^+ ^-PEP macaques that received a regimen similar to group C. Group E was one untreated V^-^Ab^± ^-PEP macaque used for SHIV_89.6P _challenge to compare the level of viral control in treated and untreated PEP-macaques. Virus infection in all macaques was evaluated using levels of plasma SIV RNA, SIV antibody responses and changes in lymphocyte subsets.

**Figure 2:**
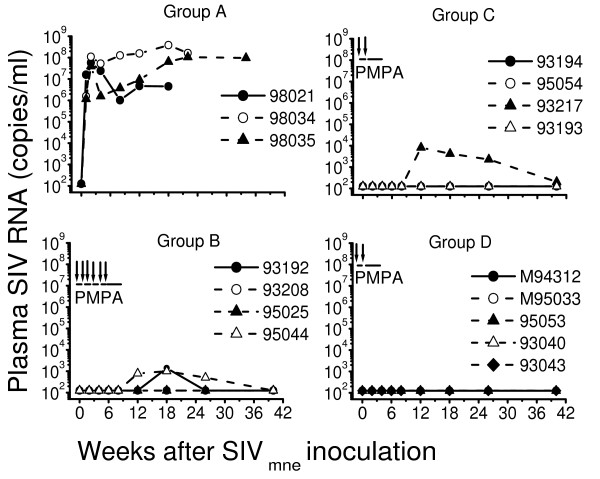
Plasma viral load levels in untreated and PMPA-treated macaques after intravenous inoculation with uncloned SIV_mne_. Group A were naïve untreated macaques. Macaques in group B (plus macaque 93043 in group D) received a 10-week PMPA treatment beginning 24 hours after virus inoculation, including treatment interruption plus SIV_mne _re-inoculation at weekly intervals during the first six weeks of treatment. Macaques in groups C and D received a 5-week PMPA treatment starting 24 hours after virus inoculation, including one treatment interruption plus SIVmne re-inoculation at week 1 of treatment. SIV RNA in plasma measurements were performed at Bayer Diagnostics (Berkeley, CA) using a branched DNA (bDNA) signal amplification assay for SIV. This bDNA assay has a lower-limit of detection of125 RNA copies/ml.

**Figure 3:**
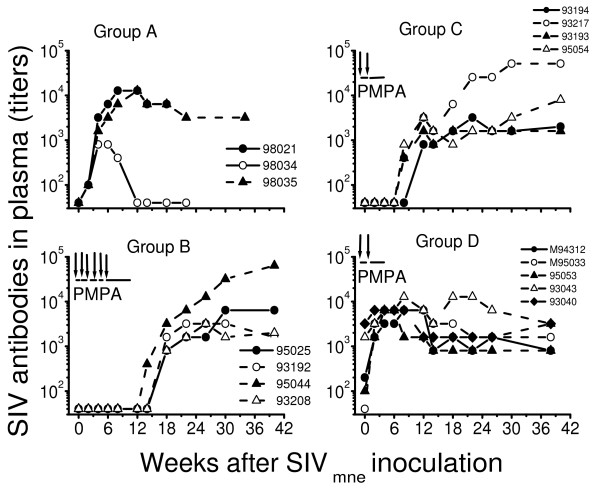
Anti-SIV IgG antibody response in untreated and PMPA-treated macaques after intravenous inoculation with uncloned SIV_mne_. Group A were naïve untreated macaques. Macaques in group B (plus macaque 93043 in group D) received a 10-week PMPA treatment beginning 24 hours after virus inoculation, including treatment interruption plus SIV_mne _re-inoculation at weekly intervals during the first six weeks of treatment. Macaques in groups C and D received a 5-week PMPA treatment starting 24 hours after virus inoculation, including one treatment interruption plus SIVmne re-inoculation at week 1 of treatment. Titers are expressed as the reciprocal of the highest dilution that was positive by HIV-2 EIA (Sanofi-Pasteur, Redmond, WA). The lowest plasma dilution used was 1:40.

All three naive, untreated macaques (group A) developed persistently high levels of plasma viral RNA within week 1 after SIV_mne _inoculation and titers of SIV antibodies by week 4, although the antibody response was lower in one macaque (macaque 98034) than in the other two untreated macaques (Fig. 2 and 3, group A).

When four V^-^Ab^- ^PEP macaques (group B) received a 10-week PMPA treatment starting 24 hours after SIV_mne _inoculation, including treatment interruptions plus SIV_mne _challenges at weekly intervals during the first six weeks of treatment, two macaques remained viremia-negative while two developed intermittent levels of plasma viral RNA beginning 2 – 8 weeks after stopping PMPA treatment. However, all the four V^-^Ab^- ^PEP macaques developed persistently high titers of SIV antibodies in plasma beginning 4–8 weeks after stopping PMPA treatment (Fig. 2 and 3, group B)

Similarly, when four V^-^Ab^- ^PEP macaques (group C) received a 5-week PMPA treatment starting 24 hours after SIV_mne _inoculation, including treatment interruption plus SIV_mne _challenge only at week 1 (i.e. after a 5-day treatment) of treatment, three macaques remained viremia-negative but one developed intermittent levels of plasma viral RNA beginning 3 weeks after stopping PMPA treatment. However, all the four V^-^Ab^- ^PEP macaques developed persistently high titers of SIV antibodies by 4 weeks after stopping PMPA treatment (Fig. 2 and 3, group C).

In contrast, when three V^-^Ab^± ^PEP macaques and two V^-^Ab^+ ^PEP macaques (group D) received a 10-week or 5-week PMPA treatment regimen similar to that for group B or C, all five macaques remained viremia-negative throughout the 26-weeks of follow-up. Moreover, they all showed immediate increase in titers of SIV antibodies plasma within 2 weeks after the initial SIV inoculation, even before PMPA treatment ended (Fig. 2 and 3, group D).

### Analysis of lymphocyte subsets

To determine whether the PMPA treatment also protected or improved the levels of CD4+ lymphocytes in peripheral blood, the absolute numbers of CD4+ lymphocytes and ratios of CD4:CD8 were analyzed for the different groups. The three naïve, untreated macaques showed a gradual decrease in CD4+ lymphocytes from 2379 ± 289/uL blood (0.987 ± 0.297 CD4:CD8 ratio) before SIV inoculation to 1240 ± 850/uL blood (0.835 ± 0.171 CD4:CD8 ratio) by 20 weeks of p.i.

The four V^-^Ab^- ^PEP macaques (group B) that received weekly treatment interruptions plus SIV_mne _challenges during the first six weeks of a 10-week PMPA treatment showed no change in the CD4+ T cells from 1648 ± 527/uL blood (CD4:CD8 ratio of 1.278 ± 0.349) before treatment to 1608 ± 969/uL blood (CD4:CD8 ratio of 1.162 ± 0.298) by 20 weeks p.i. However, the four V^-^Ab^- ^PEP macaques (group C) that received treatment interruption plus SIV_mne _challenge only at week 1 of a 5-week PMPA treatment showed gradual increase in CD4+ lymphocytes from 1589 ± 474/uL blood (CD4:CD8 ratio of 0.887 ± 0.270) before treatment to 2037 ± 408/uL blood (CD4:CD8 ratio of 1.047 ± 0.528) by 20 weeks p.i. Similarly, the five V^-^Ab^± ^or V^-^Ab^+^PEP macaques (group D) that received regimen similar to that for group B or C had gradual increase in CD4+ lymphocytes from 1523 ± 503/uL blood (CD4:CD8 ratio of 1.041 ± 0.371) before treatment to 1849 ± 753/uL blood (CD4:CD8 ratio of 1.087 ± 0.347) by 20 weeks p.i.

### CD8+ lymphocyte-suppression of viremia

**Figure 4:**
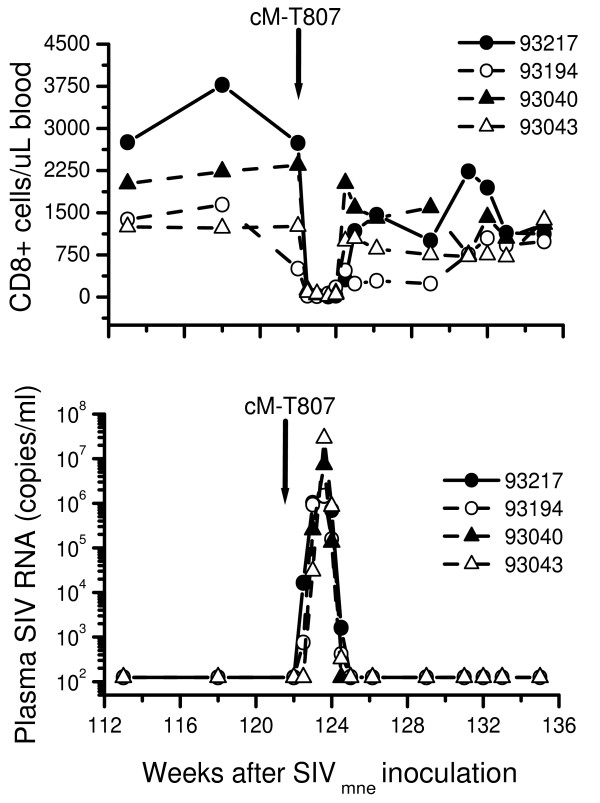
Relationship between levels of CD8+ cells in peripheral blood and plasma viral load in macaques during in vivo depletion of CD8+ lymphocytes. Depletion of CD8+ lymphocytes was achieved by using the monoclonal anti-CD8 antibody (cMT807) as described in the text. Depletion studies were performed 2.3 years after the end of PMPA-treatment. Levels of CD8+ lymphocytes were measured by flow cytometry and plasma viral load was quantitated by a branched DNA (bDNA) signal amplification assay for SIV as described in the text. Macaque 93217 and 93194 were from group C. Macaque 93040 and 93043 were from group D.

Control of viremia by CD8 T-cells has been found to be one of the major mechanisms for antiretroviral-induced host control of viral replication [18,24,25]. Therefore, depletion of CD8+ lymphocytes at several years after PMPA treatment T-cells would show both the induction and persistence of immune control of infection in the PEP macaques. Therefore, to demonstrate the presence and persistence of CD8+ lymphocyte-suppression of virus in PEP macaques, we performed *in vivo *CD8-cell depletion at 2 years after PMPA-treatment of four macaques (Fig.4). Two macaques (93194, 93217) were from group C and two (93040, 93043) were from group D. Of these four macaques, only macaque 93217 had developed detectable levels of plasma viremia before CD8-depletion. All other three macaques had no detectable plasma viremia prior to CD8-depletion. We used monoclonal antibody that depletes CD8+ T cells; including CD8+ natural killer (NK) cells [27]. After the onset of CD8+-depletion all the four macaques developed a rapid increase followed by decrease in levels of plasma viral RNA (Fig.3). Plasma viremia increased and then decreased to undetectable levels inversely with the levels of CD8+ lymphocytes-cells in peripheral blood. Moreover, the depletion of CD8+ lymphocyte-cells *in vivo *produced viremia in all macaques including those macaques that had remained negative for plasma viremia throughout the 2 years of follow-up.

### Comparison of SHIV_89.6P _challenge in treated and untreated PEP macaques

Since all the study subjects had a history PMPA PEP, one question was what the effect of pre-existing immunity alone was without the second PMPA PEP regimen. To investigate this question, we compared viral control after a pathogenic, heterologus viral challenge with SHIV_89.6P _in two PEP-macaques that were virus-negative and very weakly SIV-antibody positive after the first PMPA PEP [15], but one (M94312, group D) that received second PEP regimen and one (macaque 95020, group E) that did not. The SHIV_89.6P _– obtained originally from Dr. K.A. Riemann () – is a chimeric virus of SIV/HIV-1 containing *env *gene of HIV-1_89.6P _on SIVmac239 backbone which causes high viral loads and a rapid decline in CD4+ T-cell levels in rhesus macaques [28] and cynomogus macaques [29]. After receiving second PMPA PEP, macaque M94312 fully seroconverted and became persistently virus-negative with high level of SIV antibodies, whereas Macaque 95020 remained persistently virus-negative and weakly SIV antibody positive. Both macaques were challenged intravenously with 10 AID_50 _SHIV_89.6P _virus (6.5 years after first PMPA PEP or 2.5 years after second PMPA PEP). Two naïve macaques (macaques 99111 and 99107) served as infection controls for the SHIV_89.6P _inoculums.

**Figure 5:**
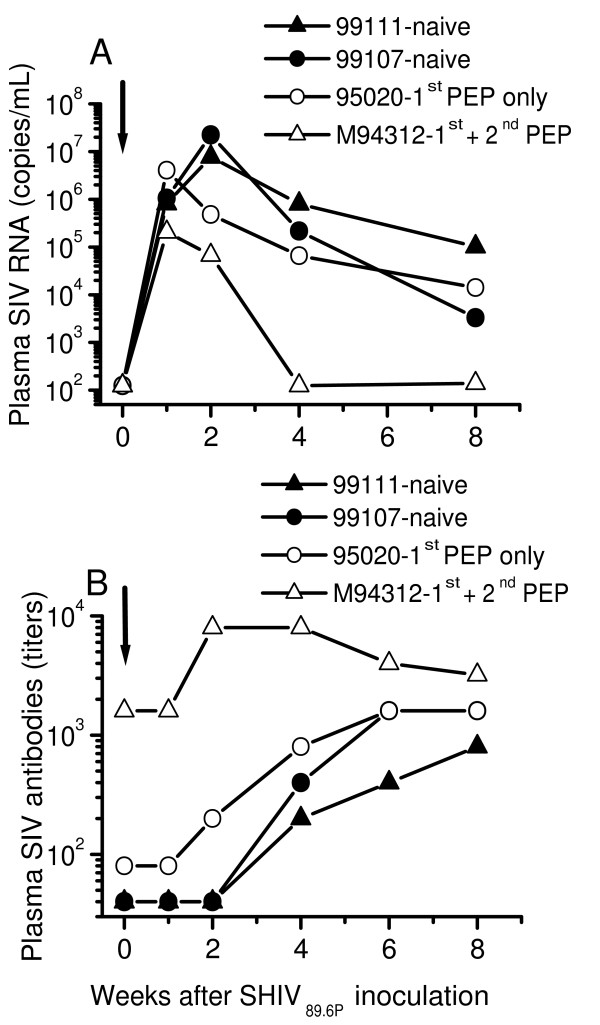
Plasma viral load (**A**) and SIV antibody response (**B**) in naïve and PEP-macaques inoculated intravenously with 10 AID_50 _chimeric SIV/HIV-1 called SHIV_89.6P_. PEP, post exposure prophylaxis. Macaques 95020 and M94312 were persistently virus-negative and weakly SIV-antibody positive (V^-^Ab^±^) after the first SIV_mne _infection/PMPA PEP regimen (1^st ^PEP) [15]. Four years later, macaque M94312 received a second PEP regimen (2^nd ^PEP) involving one treatment interruption plus SIV_mne _challenge at week 1 of a 5-week PMPA treatment. Thereafter, this macaque became persistently virus-negative and strongly-SIV antibody positive (V^-^Ab^+^). Macaque 95020 was not given a second PEP and remained persistently virus-negative and weakly-antibody positive (V^-^Ab^±^). Both macaques were then challenged with SHIV_89.6P _at the same time at 2.5 years after the 2^nd ^PEP (i.e. 6.5 years after the 1^st ^PEP). Two naïve macaques (99111 and 99107) served as infection controls. Plasma viremia was quantified by a branched DNA (bDNA) signal amplification assay for SIV by Bayer Diagnostics (Berkeley, CA). The target probes for the assay are designed to hybridize with the *pol *region of SIVmac strains of virus. This assay has a lower limit of detection of125 RNA copies/mL. Titers of SIV antibodies are expressed as the reciprocal of the highest dilution of plasma that was positive by HIV-2 EIA (Sanofi-Pasteur, Redmond, WA). The lowest plasma dilution used was 1:20.

**Figure 6:**
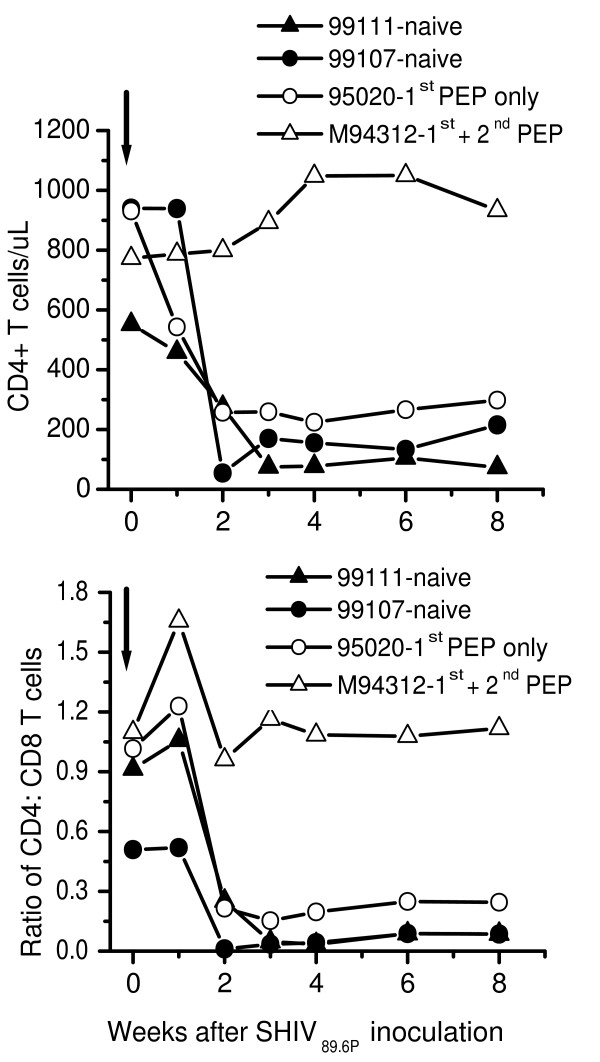
Lymphocyte subsets in naïve and PEP-macaques inoculated intravenously with 10 AID_50 _chimeric SIV/HIV-1 called SHIV_89.6P_. Figure 1 shows absolute numbers of CD4 T cells. Figure 1B shows CD4:CD8 ratio. PEP, post exposure prophylaxis. Macaques 95020 and M94312 were persistently virus-negative and weakly SIV-antibody positive (V^-^Ab^±^) after the first SIV_mne _infection/PMPA PEP regimen (1^st ^PEP) [15]. Four years later, macaque M94312 received a second PEP regimen (2^nd ^PEP) involving one treatment interruption plus SIV_mne _challenge at week 1 of a 5-week PMPA treatment. Thereafter, this macaque became persistently virus-negative and strongly-SIV antibody positive (V^-^Ab^+^). Macaque 95020 was not given a second PEP and remained persistently virus-negative and weakly-antibody positive (V^-^Ab^±^). Both macaques were then challenged with SHIV_89.6P _at the same time at 2.5 years after the 2^nd ^PEP (i.e. 6.5 years after the 1^st ^PEP). Two naïve macaques (99111 and 99107) served as infection controls. Lymphocyte subsets are analyzed in peripheral blood by FACScan for absolute number of CD4+CD3+ T lymphocytes and CD8+CD3+ T lymphocytes and used to calculate the ratio of CD4:CD8 T lymphocytes in peripheral blood.

The two-naïve macaques developed high levels of plasma SIV RNA that reached maximum (7,705,800 – 22,340,000 copies/mL) by week 2 after SHIV_89.6P _inoculation and remained persistent throughout the first 8 weeks of infection (Fig 5). Both macaques also developed a rapid decrease in peripheral blood levels of CD4+ T cells from an average of 914 ± 121 cells/μL (CD4:CD8 ratio of 0.715 + 0.2) before inoculation to persistently low levels of 75 – 150 cells/μL (CD4:CD8 ratio of 0.01 – 0.09) blood during primary infection (Fig 6). These macaques developed SIV antibody response that was detectable by HIV-2 ELISA beginning week 4 post inoculation (Fig 5).

Similarly, macaque 95020 developed high levels of plasma SIV RNA that reached a maximum of 4,067,900 copies/mL week 1 post challenge and remained persistent throughout primary infection (Fig 5). Plasma viremia was accompanied by a rapid decrease in peripheral blood levels of CD4+ T cells from 931 cells/μL (CD4:CD8 ratio of 1.016) before challenge to a persistent low levels of 220–260 cells/μL (CD4:CD8 ratio of 0.15–0.2) by week 2 post challenge (Fig 6). This macaque also became fully seroconverted with high titers of SIV antibody response detectable by HIV-2 ELISA within 4 weeks post challenge. When compared with the two naïve macaques, macaque 95020 had slightly low peak plasma viremia (4,067,900 versus 7,705,800 – 22,340,000 copies/mL) and less decline in levels of CD4+ T cells (220–260 versus 75 – 150 CD4+ T cells/uL).

In contrast, challenge of macaque M94312 with SHIV_89.6P_, resulted in transient plasma viremia that reached maximum 206,577 copies/mL at week 1 post challenge and was undetectable by week 4 post challenge (Fig 5). In addition, macaque M94312 showed no depletion of CD4+ T cells in peripheral blood; instead the CD4+ T cells increased slightly from 773 cells/μL (CD4:CD8 ratio of 1.098) at pre-challenge to 1045–1108 cells/μL (CD4:CD8 ratio of 0.961 – 1.164) between weeks 4–8 after challenge(Fig 6). This macaque also showed a transient increase in titers of SIV antibodies between weeks 2 – 6 post challenge. Thus, unlike macaque 95020 and the 2 naïve macaques, macaque M94312 controlled of SHIV_89.6P _replication within 4 weeks after inoculation and it completely blocked the depletion of CD4+ T cells from peripheral blood.

## Discussion

A 4-week, uninterrupted treatment using PMPA can completely block SIV from establishing infection in macaques if treatment is started within 24 hours after intravenous SIV inoculation, but is less effective if the initiation of treatment is delayed or if the duration of treatment is shortened [15,16]. In the present study, we show that even when treatment begins 24 h p.i. a single interruption and virus challenge was as sufficient as multiple interruptions plus viral challenges in reducing the efficacy of PMPA, instead results in persistent SIV antibody responses and long-term CD8-cell mediated suppression of virus infection. The highest efficacy showed by a 4-week, uninterrupted treatment regimen was most probably due to maintenance of effective therapeutic level of PMPA necessary to allow the infected cells initiated within 24 hours of intravenous inoculation to decay completely without spreading virus infection. However, the reduced efficacy showed by interruption of treatment plus virus challenge was most likely due to decreased PMPA levels, which allowed transient infections to occur, thereby re-setting decay of infected cells. As a result the level of SIV infection in macaques was not sufficient to establish full infection but was sufficient to induce persistent SIV antibody responses and CD8 cell-mediated suppression of virus infection. In the present study, the first interruption of treatment occurred after a 5-day treatment from the time of SIV_mne _inoculation and lasted 3 days during which macaques were re-inoculated with SIV_mne_. It is possible that, for the virus-negative and antibody negative macaques (V-Ab-PEP macaques in groups B and C, if treatment was interrupted later (e.g. on day 20 of treatment) during the 28-day treatment or if the interruption lasted less than three days (e.g. 1–2 days), the efficacy of PMPA would have been preserved due to the long intracellular half-life of the active metabolites of PMPA [26].

The findings that a majority of previously seronegative macaques developed SIV antibodies 4–8 weeks after treatment in the absence of detectable viremia or after transient viremia indicated that these macaques developed control of SIV replication by the time treatment was withdrawn, consistent with previous studies [19-23]. In addition, the findings that all the previously, weakly and strongly seropositive macaques (V^-^Ab^± ^PEP macaques) developed high titers of SIV antibodies within 2 weeks of SIV inoculation, even before the end of treatment and in complete absence of detectable viremia, indicated immune memory response [30] to SIV in these macaques. Therefore, these results indicate that the efficacy of post exposure prophylaxis with PMPA can be significantly augmented by the pre-existing immune responses to SIV. This finding is consistent with that of other investigators that showed that the efficacy of PMPA against acute or chronic SIV infection in macaques is enhanced by the presence of CD8+ T-cells [24]. PMPA itself can stimulate lymphocytes or macrophages to secrete cytokines such as tumor necrosis factor (TNF) or chemokines such as RANTES [31,32], which may have also contributed to the efficacy of PMPA.

Unlike PMPA, other antiretroviral agents such as AZT or PMEA when given in post exposure regimen are incompletely effective in blocking acute SIV infection in macaques [33-35]. The high potency of PMPA may be attributable to its rapid intracellular phosphorylation to form active metabolites, the long intracellular half-life of these active metabolites [26] and perhaps a capacity of PMPA to activate immune cells such as monocytes or lymphocytes to secrete cytokines and chemokines [31,32]. Therefore, efficacy of post exposure prophylaxis for HIV infection may depend critically not only on the timing of initiation and duration of treatment, but also on the pharmacological properties of specific antiretroviral agents used. Good candidates include highly potent antiretroviral agents such as PMPA, which are easily activated *in vivo*, have long intracellular half-life of active drug, could activate immune system and have good safety profile.

Induction of CD8 cell-suppression of viremia is one of the mechanisms by which antiretroviral treatment, including PMPA, induces host control of virus infection [13,18,24,25]. We previously demonstrated that PEP macaques exhibit considerable CD8+ lymphocyte suppression of SIV_mne _in vitro even at CD8:CD4 ratios of 1:2 and mild suppression at ratios 1:10 [17]. Our studies show that interrupted PMPA treatment resulted in CD8+ cell-suppression of viremia that persisted for more than 2 years, even in macaques that showed no evidence of viremia. (Whether macaques previously had intermittent viremia or no detectable viremia, the results were the same: plasma viral RNA increased and then decreased to undetectable levels inversely with the levels of CD8+ lymphocytes in peripheral blood). These results demonstrate the persistence of CD8+T-cell suppression of virus infection. At the same time these results demonstrate the long-term persistence of virus in the macaques, ready to replicate immediately after removal of CD8+ T cells even in macaques without any detectable virus. Such a persistence of virus in macaque may itself be a stimulant maintaining CD8 T-cell suppression of virus replication in the macaques. These results are consistent with those of other investigators showing persistence of vaccine virus as the immune correlate of protection against late onset of AIDS in macaques [36].

A previous study showed that when a 28-day PMPA PEP regimen is used against SIV_smE660 _infection in rhesus macaques, it can induce CD8+ cell-mediated control of viral replication and resistance to homologous challenge or heterologous challenge with SIV_mac39 _[18]. However, a similar regimen fails if SIV_mac239 _is used as the infecting virus in PMPA PEP [37]. Thus, the results of PMPA PEP using SIV_mne _in cynomolgus macaques in the present study may not apply necessarily to other SIV isolates or other species of macaques, or to antiretroviral regimens such as pre-exposure prophylaxis that completely block virus infection without inducing CD8+ immune responses. However, the results in the present study were obtained using SIV_mne _infection in cynomolgus macaques under specified conditions in which (i) PMPA treatment is started 24 hours after SIV_mne _inoculation, (ii) the first treatment interruption plus SIV_mne _challenge was started after a 5-day treatment, (iii) each treatment interruption lasted 3 days and macaques were challenged with SIV_mne _on the second day of interruption, and (iv) treatment was continued for at least 28 days after the last SIV_mne _challenge.

One question was how much the preexisting immunity contributed to the viral control in the PEP-macaques in the present study. This question was addressed by comparing the outcome of SHIV_89.6P _challenge in two PEP-macaques (95020 and M94312) that had similar outcomes from first PMPA PEP, but one macaque M94312 that received second PMPA PEP regimen whereas one (macaque 95020) did not. After the initial PMPA PEP both macaques had no detectable viremia, but they had very weak SIV-specific antibody response. Four years later, macaque M94312 was given second PEP regimen and thereafter became persistently virus-negative but strongly positive for SIV antibody response. When challenged intravenously with 10 AID_50 _SHIV_89.6P. _macaque 95020 failed to control active viral replication and depletion of CD4+ T cells throughout the course of primary infection. In contrast, macaque M94312 completely controlled viral replication within 4 weeks of inoculation and completely blocked the depletion of CD4+ T cells. Although the number of macaques in this challenge study was small, the marked viral control by M94312 in contrast to macaque 95020 was most probably a contribution of second PMPA PEP regimen. It is conceivable that the individual PEP-macaques or macaque groups such as V^-^Ab^-^, V^-^Ab^± ^or V^-^Ab^+ ^had different levels of pre-existing SIV specific CD8+ cell responses which contributed to the protection of macaques independent of PMPA. Van Rompay et al [24] demonstrated that CD8+ T cells enhanced the efficacy of PMPA treatment in controlling SIV infection. Therefore, in the presence of PMPA treatment, preexisting immune response might interact additively with PMPA to control infection. The outcome would depend on the level of pre-existing immunity [14]. Although our study has a limitation in its capacity to establish the absolute contribution in each macaque group, it provides an insight into relative contribution to efficacy by comparing the results of efficacy between macaque groups. For example, our findings show that all the seronegative (V^-^Ab^-^) PEP macaques in groups B and C seroconverted only when PMPA treatment had stopped for at least 4 – 8 weeks, irrespective of 5-week or 10-week duration of treatment. Similarly, the onset of transient or intermittent viremia in the three macaques in those groups also developed 2 – 8 weeks after stopping PMPA treatment. In contrast the macaques in group D that received a similar treatment regimen, but which were previously weakly seroposotive, became fully seroconverted within two weeks of SIV inoculation, even before the end of PMPA treatment. These findings suggest strongly that, viral control was more dependent on PMPA treatment in the previously seronegative PEP macaques than in the previously weakly or strongly seropositive PEP macaques.

Overall, our studies confirm that early initiation of potent antiretroviral treatment and strict compliance to the duration of treatment are critical factors for the success of post exposure prophylaxis. The initiation of PMPA treatment during chronic SIV infection fails to control the rebound of viremia after treatment is withdrawn [38]. However, the initiation of PMPA treatment between 2 days to 6 weeks after SIV inoculation (i.e. during the primary phase of acute SIV infection) results in partial immune control of SIV rebound [19-23]. Yet, for a preventive treatment against HIV to be effective, the treatment must block the HIV from ever establishing systemic infection in the lymphoid organs [39]. We have found that the most effective regimen for blocking SIV infection in macaques is a 4-week, uninterrupted treatment using PMPA beginning before or within 24 hours of SIV intravenous inoculation [15,16]. Our study shows that if the PMPA treatment is interrupted and macaques are re-exposed to virus, then the complete protection from SIV infection is abrogated. Despite the lack of complete protection, a majority of macaques still benefit by developing long-term immune control of virus infection. More over the use of structured treatment interruptions of highly active antiretroviral therapy in HIV-infected persons [13,25] or in SIV-infected macaques [22] has been shown to induce the immune control virus infection. The effectiveness of such regimens depends on early initiation of treatment, the potency of the antiretroviral drug, and the immune responsiveness of the host [14]. In addition, a recent clinical trial in human found that under controlled conditions of CD4-guided treatment, scheduled treatment interruptions may also be beneficial in reducing the total amount of antiretroviral drug used, and thus reduce cost as well as prevent drug toxicity without the risk of developing drug resistance and loss of efficacy of treatment [40]

## Conclusion

In order for the regimen of post exposure prophylaxis to be effective in preventing HIV transmission in persons exposed to HIV in blood or body fluids from HIV-infected persons, the exposed individuals need to comply with the prescribed regimen of potent antiretroviral treatment started as early as possible. However, in cases of noncompliance to treatment regimen, our study shows that there is still a benefit to completing treatment because interrupted regimens can reduce the possibility of an established persistent viral replication and instead modifies the kinetics of viral replication to induce immune control of the infection. Our study also implies that post-exposure regimens of PMPA treatment in the SIV/macaque is a useful animal model for inducing long-term immune control of virus replication and thus useful for investigating the nature of protective immune responses and strategies of therapeutic vaccination or immune boosting against HIV.

## Materials and methods

### Macaques

The study subjects, henceforth called post-exposure prophylaxis (PEP) macaques, were fourteen Cynomolgus macaques (*Macaca fascicularis*) that had been previously inoculated intravenously with SIV_mne_, then treated with PMPA or PMEA [15-17] and evaluated for 4 to 5 years (Table 1). Three naïve macaques served as controls for infectivity of SIV_mne _(group A). Eight of the PEP-macaques, called V^-^Ab^- ^PEP-macaques (groups B and C), were completely protected from infection since they had no detectable virus or SIV antibodies throughout the 4 – 5 years of follow-up. Four PEP-macaques (three are in group D and one is in group E), called V^-^Ab^± ^PEP-macaques, had transient infection early after treatment and thereafter were free of virus but developed weak SIV antibody response (ELISA titers less than 1:200, and 1–2 bands on SIV immunoblot). Two PEP-macaques, called V^-^Ab^+ ^PEP-macaques (in group D), had been completely protected by treatment but when challenged with a highly pathogenic, heterologous virus (SIV_smmPBj14_), they resisted the challenge and became persistently positive for SIV antibodies [17]. Care and husbandry of all macaques were in strict conformance with federal guidelines. All procedures were approved by the Institutional Animal Care and Use Committee at the University of Washington.

### Virus inoculum

The SIV_mne _stock used in the second PMPA PEP regimens was derived from the supernatant of uncloned SIV_mne _propagated in HuT 78 cells and expanded in macaque PBMCs [15,16]. One milliliter of the stock contained 10,000 viral particles of 50% tissue culture infectious doses (TCID_50_) as determined in human T-cell lines or 1,000 50% animal infectious doses (AID_50_) as determined by intravenous inoculation of macaques, or100 AID_50 _as determined by intrarectal inoculation of macaques. The SHIV_89.6P _stock used in the post PEP challenge was originally obtained from Dr. K. A. Reimann (Beth Israel Hospital, Boston MA, Reimann et al 1996) and propagated in phytohemagglutinin-A-stimulated PBMCs from naïve pigtailed macaques [40]. One milliliter of the stock contained 10,000 TCID_50 _as determined in MT-2 cells or 1,000 AID_50 _by intravenous inoculation in macaques.

### Regimen of post-exposure prophylaxis

The PMPA PEP regimen used in this study is illustrated schematically in Figure 1, to show the schedule of SIV_mne _inoculations and treatment interruptions for different groups. Three naïve macaques were used as untreated control (group A). Fourteen PEP macaques were divided into groups based on the level of their pre-existing SIV antibody response. The eight V^-^Ab^- ^PEP-macaques were divided into two groups (B and C) of four macaques each. Three V^-^Ab^± ^PEP-macaques and two V^-^Ab^+ ^PEP-macaques formed group D. One V^-^Ab^± ^PEP-macaque (95020) formed group E. All the macaques in groups A – D were first inoculated intravenously with 1AID_50 _(10 TCID_50_) of SIV_mne _on day 0 and then reinocluated intravenously 7 days later with 10 AID_50 _(100 TCID_50_) SIV_mne_. All PEP-macaques in groups B to D were started on PMPA treatment (30 mg/kg per day, subcutaneously) beginning 24 hours after initial intravenous SIV inoculation. After 5 days of daily dosing, treatment was interrupted for 72 hours while macaques were re-inoculated intravenously with 10 AID_50 _SIV_mne_. Then daily dosing resumed 24 hours after re-inoculation for additional 28 days. However, all macaques in group B and one macaque (93043) in group D received additional interruptions of PMPA-treatment plus re-challenge with SIV_mne _for six weeks. For these macaques, treatment was interrupted for 72 hours while macaques were re-inoculated intrarectally with 10 AID_50 _(1, 000 TCID_50_) SIV_mne _at weekly intervals until week 6 after initial SIV_mne _inoculation. Thereafter, they all resumed daily dosing for additional 28 days.

### Depletion of CD8+ T cells *in vivo*

cM-T807 anti-CD8 monoclonal antibody was received from Maine Biotechnology Services (Portland, ME 04103) and Covance Research Products Inc. (Denver, PA 17517). At 120 weeks from the beginning of the study, we performed *in vivo *depletion of CD8 cells in four macaques (93194, 93040, 93043 and 93217) using monoclonal antibody (anti-CD8 cM-T807) as described by Schmitz et al 1999 [27].

### SHIV_89.6P _challenge

To investigate the relative contribution of the second PEP regimen to viral control, we performed SHIV_89.6P _challenge on two PEP-macaques (95020 and M94312) that had similar outcomes of being persistently V^-^Ab^±^after the first PMPA PEP, but one (M94312) received second PMPA PEP regimen while the other (95020) did not. As described previously [15] macaque M94312 had previously been given PMPA treatment for 28 days beginning 24 hours after intravenous SIV_mne _inoculation. Macaque 95020 was given PMPA treatment for only 10 days beginning 24 hours after SIV_mne _inoculation. Both macaques had no detectable viremia (plasma SIV RNA or infectious cells in peripheral blood), but they developed weak SIV-antibody response beginning 24 weeks post inoculation detectable as a very weak band for SIVgp120 on immunoblotting (Table 1). Four years later macaque M94312 was given second PMPA PEP regimen of a 5-week PMPA treatment involving a single treatment interruption plus SIV_mne _challenge at week 1 of treatment. Thereafter, this macaque became virus-negative but strongly SIV-antibody positive (V^-^Ab^+^) (Fig 1, 2, 3). Macaque 95020 was not given second PMPA PEP and remained persistently V^-^Ab^± ^(virus-negative and weakly-seroposoitive). In the present study, we challenged both macaques intravenously with10 AID_50 _SHIV_89.6P _(6.5 years after first PMPA PEP or 2.5 years after second PMPA PEP). Two naïve macaques (macaques 99111 and 99107) served as infection controls for the virus inoculum. Infection was evaluated during the first 8 weeks after inoculation. The SHIV_89.6P _– was obtained originally from Dr. K.A. Riemann and expanded in macaque PBMC. Its is a chimeric virus of SIV/HIV-1 containing *env *gene of HIV-1_89.6P _on SIVmac239 backbone which causes high viral loads and a rapid decline in CD4+ T-cell levels in rhesus macaques [28] and cynomogus macaques [29]. Plasma SHIV RNA was determined by branched DNA (bDNA) signal amplification assay for SIV, lymphocyte subsets by FACScan flow cytometry (Becton Dickinson, San Jose, CA) for specific CD4+, CD8+, CD3^+ ^and CD20^+ ^lymphocyte subsets. SHIV-antibody response was quantified by limiting dilution assay using a commercial HIV-2 EIA (enzyme immunoassay) kit (Sanofi-Pasteur, Redmond, WA) as described previously [29,41].

### Clinical observations

All macaques were observed daily for general physical condition including appetite, stool consistency, and demeanor. At predetermined time points during the 144 weeks of study, the animals were anesthetized with ketamine for thorough physical examination including body weight and temperature. Blood was drawn for complete blood count, serum chemistry, viriology, and lymphocyte subset analyses. Data from clinical assessments was used to monitor the course of SIV infection and clinical disease including drug toxicity.

### Laboratory assays

Laboratory determinations included plasma viral RNA, titers SIV-antibodies and CD4+ and CD8+ T cell counts. Quantitative assays for viral RNA in plasma were performed at Bayer Diagnostics (Berkeley, CA) using a branched DNA (bDNA) signal amplification assay for SIV. This bDNA assay has a lower-limit of detection of125 RNA copies/ml [39]. Titers of SIV antibodies were determined by end-point dilution of plasma using HIV-2 EIA kit (Sanofi-Pasteur, Redmond, WA). Lymphocyte subsets in EDTA-blood were determined by FACscan using mouse anti-human monoclonal antibodies that react with macaque lymphocytes to determine the absolute numbers of CD4+ and CD8+ T cells and the CD4:CD8 cell ratio was calculated [41].

## Competing interests

The author(s) declare that they have no competing interests.

## Authors' contributions

PE carried out the experiments, performed data analysis and wrote the initial drafts of the manuscript, YJ helped with laboratory experiments and data interpretation, MA helped with experimental design and data interpretation, BT helped with laboratory and animal experiments and data analysis, GB assisted with animal experiments. CCT was responsible for the overall experimental design, data interpretation and implementation of the project.
